# On the Fœtus in Utero

**Published:** 1886-06

**Authors:** 


*On the Fœtus in Utero.* In a series of Essays by ALEXANDER HARVEY, M.D. Pp. 140. London: H. K. LEWIS.

Five essays bearing on the interesting subject of Fœtal Inoculation, and printed in various periodicals some years ago, are here gathered together in a little volume. The essays are scientific and scholarly, exceedingly suggestive, and written in elegant language. The writer is very chary in drawing conclusions, but he presents his facts in sequences which cannot fail to suggest inferences. The whole subject is well worthy of careful study, and Dr. Harvey has done well in gathering together these numerous observations, and presenting them so clearly and graphically to the student.

